# Lipid Peroxidation Products in Human Health and Disease

**DOI:** 10.1155/2013/583438

**Published:** 2013-11-02

**Authors:** Kota V. Ramana, Sanjay Srivastava, Sharad S. Singhal

**Affiliations:** ^1^Department of Biochemistry and Molecular Biology, University of Texas Medical Branch, Galveston, TX 77555, USA; ^2^Environmental Cardiology, University of Louisville, Louisville, KY 40202, USA; ^3^Department of Diabetes and Metabolic Diseases Research, Beckman Research Institute, City of Hope National Medical Center, Duarte, CA 91010, USA

Enhanced formation of reactive oxygen species (ROS) leads to tissue dysfunction and damage in a number of pathological conditions. ROS oxidize the lipids to generate peroxides and aldehydes. These lipid peroxidation products, especially oxidized lipids-derived aldehydes, are much more stable than the parent ROS and therefore can diffuse from their site of generation and inflict damage at remote locations. Therefore, products of lipid oxidation can extend and propagate the responses and injury initiated by ROS. The lipid peroxidation (LPO) products are highly reactive and display marked biological effects, which, depending upon their concentration, cause selective alterations in cell signaling, protein and DNA damage, and cytotoxicity. Increased formation of lipid peroxides and aldehydes has been observed in atherosclerosis, ischemia-reperfusion, heart failure, Alzheimer's disease, rheumatic arthritis, cancer, and other immunological disorders. Therefore, decreasing the formation of lipid peroxidation products or scavenging them chemically could be beneficial in limiting the deleterious effects of ROS in various pathological conditions. Indeed, recent studies have identified several agents that could interfere with the LPO-mediated cell signaling pathways and could act as potential therapeutic drugs. 

 This special issue on LPO products in human health and disease compiles 20 excellent manuscripts including clinical studies, research articles, and reviews, which provide comprehensive evidence demonstrating the significance of LPO products in various pathological conditions. 

The five review articles of this issue describe current knowledge of oxidative stress induced inflammatory signaling and inflammatory complications. The review article by C. Signorini et al. described the role of specific LPO products in the clinical manifestations and natural history of Rett syndrome, a genetically determined autism spectrum disorder. Specifically, they described how LPO-derived aldehyde products such as 4-hydroxynonenal (4-HNE) and a series of isoprostanes compounds could act as biomarkers of the disease. The review article by S. Joshi et al. described the factors involved in oxalate and calcium-oxalate induced injury in the kidneys and demonstrated how oxidative stress generated ROS via NADPH oxidase could contribute to renal injury. Further, they discussed, in depth, the pathophysiological role of NADPH oxidase in the kidneys and how different antioxidants and NADPH oxidase inhibitors could be used to control hyperoxaluria-induced kidney stone diseases. S. E. Lee and Y. S. Park in their review article discussed how the most potent LPO-derived *α*, *β*-unsaturated aldehydes such as acrolein, 4-HNE, and crotanaldehyde were involved in the pathophysiology of vascular complications. Specifically, the authors have nicely described the molecular mechanism by which *α*, *β*-unsaturated aldehydes cause vascular inflammation and dysfunction. The review article by P. Filipe et al. discussed how ultraviolet B- (UVB-)induced alterations in the proteins of the interstitial fluid may make consequential contributions to inflammation and degenerative processes of skin under UVB exposure. Further, this article highlights the significance of LPO, high-density lipoprotein (HDL), and low-density lipoprotein (LDL) as important targets of UVB in the interstitial fluid. Final review article by U. C. S. Yadav and K. V. Ramana discussed the significance of LPO-derived lipid aldehydes in the regulation of nuclear factor-kappa binding protein (NF-*κ*B)-induced inflammatory cell signaling. Specifically, they described how aldose reductase catalyzed lipid aldehydes such as 4-HNE and acrolein and their glutathione-conjugates act as secondary singling molecules to activate redox sensitive transcription factor NF-*κ*B mediated inflammatory signals to contribute to inflammatory pathologies.

The research article by B. A. Leon-Chavez et al. investigates the effect of nitric oxide inhibitor, L-NAME, and zinc on nitrosative stress and cell death in a transient ischemia model by common carotid-artery occlusion in rats. In this study, administration of rats with ZnCl_2_ reduces accumulation of zinc, level of nitrite and LPO, and cell death in the late phase of the ischemia. Fascinatingly, L-NAME administration in the rats treated with zinc causes time dependent increase in the accumulation of zinc in the early phase and increase in the levels of nitrites, LPO, and cell death by necrosis in the late phase. Based on the results, authors conclude that co-administration of zinc along with L-NAME increased the injury caused by common carotid-artery occlusion when compared to administration of zinc alone. The article by N. S. N. Yusoff et al. examines how antihypertensive drug clonidine regulates oxidative stress in rats. Their study indicates that clonidine in addition to its hypotensive effect enhances the level of antioxidant status and ameliorates the oxidative stress which could reduce the hypertension-induced heart damage in spontaneously hypertensive rats without or with L-NAME administration. Studies by P. Fernández-Robredo et al. demonstrate the effect of lutein and a multivitamin complex with lutein and glutathione on systemic and retinal biochemical and ultrastructural parameters leading to AMD progression in the apoE null mice. These studies indicate that treatment of lutein along with multivitamin complex but not lutein alone substantially reduced VEGF levels and MMP-2 activity and ameliorated the retinal morphological alterations in the apoE null mice. 

The research article by S. Sahreen et al. investigates the effect of extracts of Rumex hastatus roots on carbon tetrachloride-induced hapato and testicular oxidative stress and toxicity in the rats. The results provide some evidence that the extracts of Rumex hastatus roots could have beneficial antioxidant properties in preventing free radical-mediated toxicities. In the article by A. Ścibior et al., the protective effects of magnesium against vanadium-induced LPO in rat hepatic tissues were examined. This is an interesting report on how the combination of metal treatments regulates cellular oxidative stress *in vitro* and *in vivo* conditions. I. Sano et al. in their article reported the pathological mechanism of pterygia. Specifically, they have shown the involvement of protein adducts of LPO-derived aldehydes such as 4-HNE and 4-hydroxyhexenal (4-HHE) in human pterygia patient's eyes. 

The relation between fluoride intake and oxidative stress in salivary glands of rats was reported by P. M. Yamaguti et al. in their article. Especially, they demonstrate that the single administration of sodium fluoride in rats decreased super oxide dismutase while increased the LPO in the salivary glands as early as in four hours. Interestingly, the oxidative stress induced by sodium fluoride is more in submandibular glands as compared to parotid glands. H.-M. Luo et al. in their research article examined the effects of ulinastatin on burned swine by monitoring the hemodynamic variables by PiCCO system and determining the extent of LPO and tissue edema. Results indicate that LPO regulates ulinastatin diminished burn-induced increase in vascular permeability and net fluid accumulation.

This study suggests a potential small-volume fluid resuscitation strategy in combating with a major burn injury. In the final research article of this issue, Z. Yiran et al. reported involvement of oxidative stress and MAPK in cadmium-induced hepatic cell toxicity and apoptosis. Preincubation of N-acetyl-L_cysteine with BRL3A liver cells decreased cadmium-induced increase in the malondialdehyde level, super oxide dismutase (SOD) and glutathione peroxidase activity and cell viability. The involvement of cadmium-induced hepatotoxicity was confirmed by using inhibitors of p38 MAPK, JNK, and ERK.

The exciting clinical study reported by C. Cipierre et al. indicates malondialdehyde adduct to hemoglobin as a novel biomarker of oxidative stress in the preterm neonates. By using LC-MS/MS methods, this study identified formation of malondialdehyde adducts of hemoglobin from the blood of full-term and preterm neonates and based on these findings authors suggest that the malondialdehyde adduct of hemoglobin might be a useful marker for variety of pathological conditions. In the same issue, C. Cipierre et al. also assessed the influence of the perinatal condition on malondialdehyde-hemoglobin adduct concentrations. In this pilot clinical study, authors found a relationship between blood malondialdehyde-hemoglobin adduct concentrations with neonatal morbidity in very low birth-weight infants. Results from these two manuscripts indicate the significance of early antioxidant treatment to decrease oxidative stress mediated problems in very low birth-weight infants. 

Interconnections between the pathological conditions associated with the diabetes and cirrhosis patients were reported by R. Hernández-Muñoz et al. in a clinical study. This study indicates that the levels of oxidative stress markers in the red blood cells of cirrhotic patients were significantly increased, whereas in the patients with coincidence of diabetes and cirrhosis oxidative response was partially reduced. Interestingly, the levels of total phospholipids and cholesterol were enhanced in the patients with both pathologies but not in the patients with the single pathology. In another clinical study, D. Miric et al. have examined the involvement of xanthine oxidase (XOD) in oxidative damage and inflammatory response in end-stage renal disease (ESRD) patients. Results show that patients suffering from ESRD had higher levels of oxidative stress markers such as hydroperoxides, AOPP, SOD, and XOD and a lower level of total SH groups when compared to healthy subjects. Interestingly, XOD activity was only increased in patients with poor nutritional status as indicated by geriatric nutritional risk index (GNRI) score. These findings suggest that increased XOD may contribute to oxidative injury during hemodialysis treatment leading to the pathogenesis of malnutrition-inflammation complex syndrome.

H. Tuzcu et al. have reported the effect of high-dose insulin analog initiation therapy on glycemic variability, LPO, and oxidative stress determined in the plasma and urine samples of type 2 diabetes patients. Especially, treatment with insulin analog along with metformin resulted in a significant reduction in glycemic variability and oxidative stress markers when compared to single oral hypoglycemic agent. Although this study was carried out in 24 patients, the data indicates that the treatment with insulin analogs, regardless of blood glucose changes, exerts inhibitory effects on LPO. In another clinical study, C. Mila-Kierzenkowska et al. have identified the effect of radical nephrectomy on LPO and redox balance in the patients with renal cell carcinoma. The findings suggest that laparoscopy may be used for radical nephrectomy as effectively as open surgery without creating any oxidative stress in the patients of renal cell carcinoma.

 In a final note, it is evident from these papers that LPO products play a major role in deregulating key pathways involved in the pathophysiology of a number of pathological disorders. The treatment options that control the production of free radical-mediated generation of LPO products could be potential therapeutic approaches to controlling human diseases. 

